# Protein and DNA synthesis demonstrated in cell-free *Ehrlichia chaffeensis* organisms in axenic medium

**DOI:** 10.1038/s41598-018-27574-z

**Published:** 2018-06-18

**Authors:** Vijay K. Eedunuri, Yuntao Zhang, Chuanmin Cheng, Li Chen, Huitao Liu, Anders Omsland, Dan Boyle, Roman R. Ganta

**Affiliations:** 1 Center of Excellence for Vector-Borne Diseases, Department of Diagnostic Medicine/Pathobiology, College of Veterinary Medicine, Kansas State University, Manhattan, KS 66506 USA; 2 Paul G. Allen School for Global Animal Health, Washington State University, Pullman, WA 99164 USA; 3 Division of Biology, Kansas State University, Manhattan, KS 66506 USA; 4 Present Address: UT Health Science Center at San Antonio, San Antonio, TX USA; 5 Present Address: Vanderbilt University Medical Center, Nashville, TN USA

## Abstract

*Ehrlichia chaffeensis*, a tick-transmitted rickettsial bacterium, is the causative agent of human monocytic ehrlichiosis. Biochemical characterization of this and other related Rickettsiales remains a major challenge, as they require a host cell for their replication. We investigated the use of an axenic medium for *E*. *chaffeensis* growth, assessed by protein and DNA synthesis, in the absence of a host cell. *E*. *chaffeensis* organisms harvested from *in vitro* cultures grown in a vertebrate cell line were fractionated into infectious dense-core cells (DC) and the non-infectious replicating form, known as reticulate cells (RC) by renografin density gradient centrifugation and incubated in the axenic medium containing amino acids, nucleotides, and different energy sources. Bacterial protein and DNA synthesis were observed in RCs in response to glucose-6-phosphate, although adenosine triphosphate, alpha-ketoglutarate or sodium acetate supported protein synthesis. The biosynthetic activity could not be detected in DCs in the axenic medium. While the data demonstrate *de novo* protein and DNA synthesis under axenic conditions for *E*. *chaffeensis* RCs, additional modifications are required in order to establish conditions that support bacterial replication, and transition to DCs.

## Introduction

*Ehrlichia chaffeensis* is an obligate intracellular, tick-transmitted bacterium that is maintained in nature in a cycle involving at least one and perhaps several vertebrate reservoir hosts^1,2^. Human infections with *E*. *chaffeensis* cause the disease human monocytic ehrlichiosis (HME) which is characterized by an acute onset of febrile illness that can progress to a fatal outcome, particularly in immune compromised individuals, elderly and children^3,4^. People undergoing blood transfusions and organ transplantations are also at high risk in acquiring *E*. *chaffeensis* infections and HME^5,6^. Knowledge of the biology and natural history of *E*. *chaffeensis*, and of the epidemiology, clinical features, and laboratory diagnosis has expanded considerably since its discovery^7–10^.

The life cycle of *E*. *chaffeensis* involves a tick vector and a mammalian host. In both mammalian and tick cells, *E*. *chaffeensis* transitions between two forms; the smaller dense-core cells (DCs) with dense nucleoid and the larger pleomorphic reticulate cells (RCs). RCs possess uniformly dispersed nucleoid filaments and ribosomes, sometimes forming long projections of the cell wall, protrusions of the cytoplasmic membrane into the periplasmic space, or budding protoplast fragments into the periplasmic space^11–13^. DCs are considered the infectious form of the bacterium, which enter naïve host cells by phagocytosis, then transform to non-infectious RCs within a phagosome and replicate prior to retransforming and releasing as DCs from the cells. We are yet to understand the detailed differences in proteins expressed in the two distinct forms of *E*. *chaffeensis* in vertebrate and tick cells and how the entire process is regulated.

Recent advances with *Coxiella burnetii* demonstrate that axenic media aids greatly in studies focused on biochemical and genetic analysis of the pathogen^14–19^. Similarly, the use of axenic media for protein biosynthesis has been reported for *Chlamydia trachomatis*, although bacterial replication is yet to be demonstrated^20,21^. These studies suggest that it is possible to take advantage of the cell-free growth medium for other important obligates belonging to Rickettsiales, such as *E*. *chaffeensis*. An axenic growth medium, called acidified citrate cysteine medium (ACCM), supported about three logs of growth of *C*. *burnetii* over 6 days in a microaerobic environment^14^. The ability to grow obligate intracellular bacteria under axenic conditions represents a major advancement, as it will enable new paths of investigation, such as aiding the manipulation of the pathogenic organisms in the absence of host cells, clonal purification of bacterial mutants, and detailed biochemical and genetic studies. Indeed, its application is well documented in greatly advancing the research on *C*. *burnetii*^15^. However, axenic growth methods require considerable optimization to adapt to each obligate pathogen of interest.

The purpose of this study is to evaluate the possibility of developing axenic culture methods for *E*. *chaffeensis*. In this study, we attempted to use the medium previously described for *C*. *trachomatis*, referred to as CIP-1^20^. We describe the use of CIP-1 in supporting both protein and DNA biosynthesis in DC and RC forms of *E*. *chaffeensis* in the absence of host cells.

## Results

### Protein synthesis in cell-free *E. chaffeensis* assessed in axenic media

**Figure 1:**
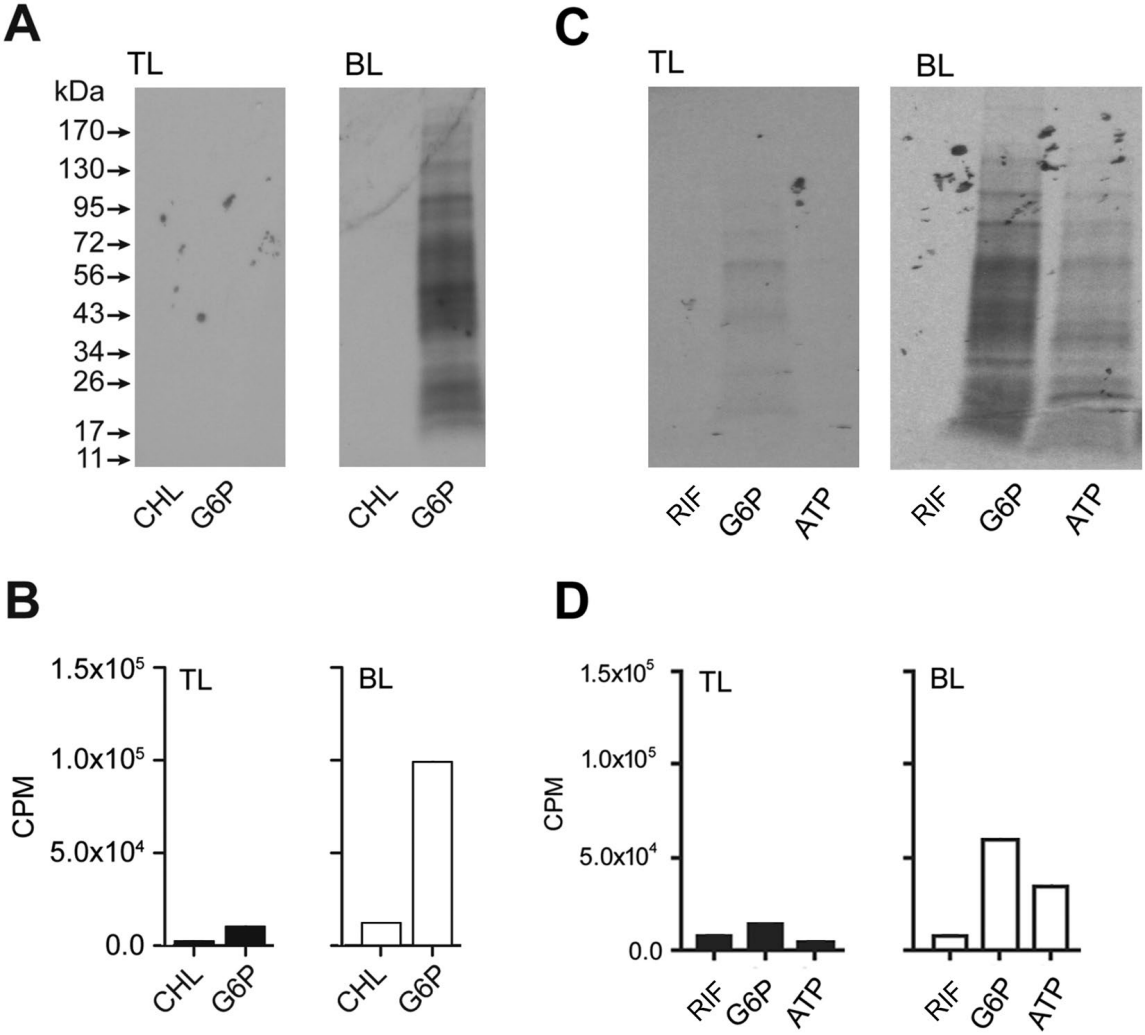
Incorporation of ^35^S Cys-Met into *E*. *chaffeensis* organisms recovered from renografin fractionated bacteria in the axenic medium. (**A**) *E*. *chaffeensis* organisms isolated from Vero cells used in the axenic media assessment; autoradiography image assessing the incorporation of ^35^S Cys-Met in *E*. *chaffeensis* recovered from renografin purified top (TL) and bottom (BL) layered fractions incubation in the axenic media with or without chloramphenicol (CHL) and with G6P as an energy source and incubated for 24 h (G6P) (**B**). As in panel A, but quantitation of radiolabel incorporation by scintillation count analysis data (**C**). As in panel A except that the organisms were recovered from infected HL60 cells; this experiment also included a fraction of the purified organisms incubated in the axenic media with ATP as the energy source (ATP) (**D**). As in panel C, but the scintillation counting data were presented.

The axenic medium used for *C*. *trachomatis* is a complex mixture containing intracellular phosphate buffer (IPB) supplemented with 1% FBS, 25 μM of equimolar mix of all 20 amino acids (AA), 0.5 mM glucose 6-phosphate (G6P), 1.0 mM ATP, 0.5 mM DTT, and 50 μM each of GTP, UTP, and CTP as described in^20^, except when using alpha ketoglutarate (0.5 mM) or sodium acetate (0.5 mM) as the carbon source. When assessing ^35^S Cys-Met incorporation, concentrations of these two cold amino acids were reduced to 1 μM and then the two radioactive amino acids were supplemented in the form of 70 µCi of ^35^S-Cys-Met. Protein synthesis in the axenic media is verified with inclusion of chloramphenicol (CHL) or rifampin (RIF). In the current study, we prepared the medium with or without an energy source and used it to determine if it supports *E*. *chaffeensis* protein synthesis in the absence of a host cell. *E*. *chaffeensis* dense-core cells (DCs) and reticulate cells (RCs) were purified from the infected Vero cells or DH82 cells by renografin gradient centrifugation which fractionated at the interface between PBS/25% renografin (top layer) and between 25–35% renografin fraction (bottom layer), respectively. Incubation of *E. chaffeensis* purified from the bottom layer of the renografin fraction, where the RCs appeared to have concentrated, resulted in detectable protein synthesis. Protein synthesis by bacteria isolated from the top fraction, which is likely to contain DCs, was comparably much lower (Fig 1). Protein synthesis was abolished when CHL was included in the media and similarly with the inclusion of RIF (Fig. 1).

### Defining DCs and RCs in the renografin fractions

**Figure 2:**
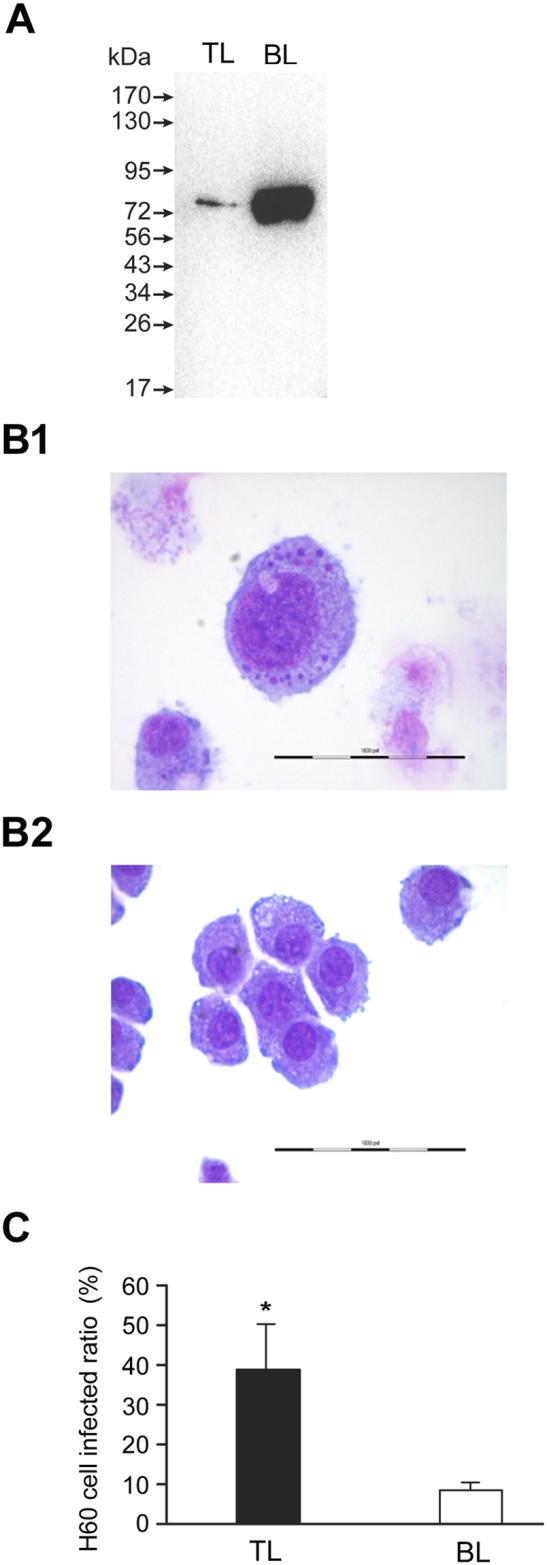
The presence of DCs and RCs assessed in the TL and BL of the renografin purified *E*. *chaffeensis* fractions. (**A**) Western blot analysis performed with ClpB polyclonal antibody which revealed higher levels of the protein expression in the BL derived bacterial fraction proteins; (**B**) cell-free *E*. *chaffeensis* recovered from TL and BL assessed for reinfection of naïve Vero cells; infection was detected only with the fraction derived from the TL (**B1**), but not in the BL (**B2**) (**C**). Infectivity of fractionated *Ehrlichia* organisms from the TL and BL was further confirmed by measuring the numbers of infected cells following incubation for three days following inoculation into naïve HL60 cultures. Infectivity with TL-derived *Ehrlichia* was significantly more than BL derived bacteria.

**Figure 3:**
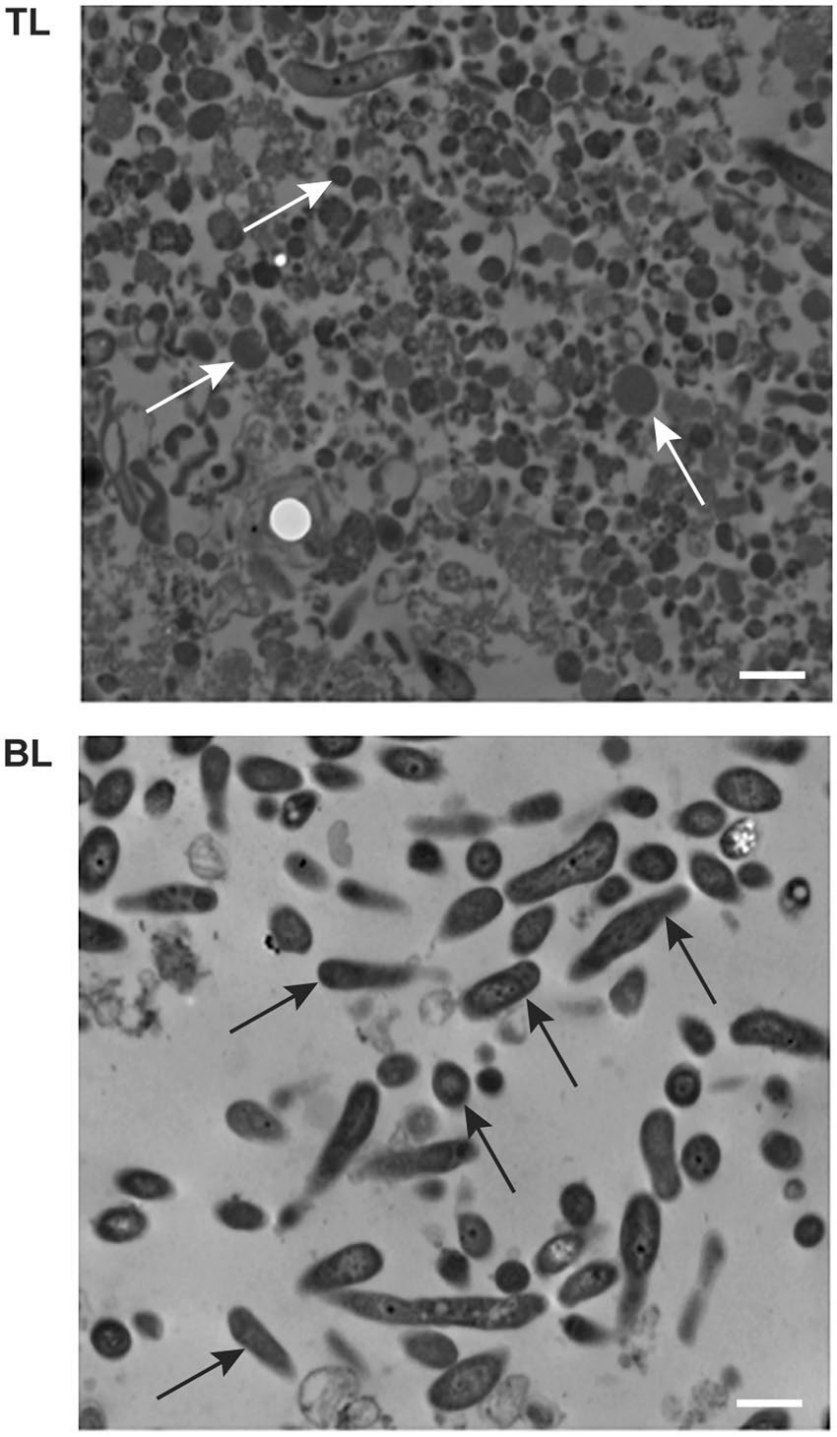
Transmission electron microscopy (TEM) analysis to define the *E*. *chaffeensis* organisms present in TL and BL. TEM images of the top (**A**) and bottom (**B**) layers where prototypical small DCs and pleomorphic RCs of *E*. *chaffeensis* were observed, respectively, throughout the images (a few DC and RC are identified with arrows). (Scale bars = 1 μm).

The presence of DCs and RCs in the top and bottom layers of the renografin-purified fractions, respectively, was confirmed by Western blot analysis, *in vitro* infection assessment (Fig. 2) and transmission electron microscopy (Fig. 3). We previously reported the enhanced expression of *E*. *chaffeensis* ClpB in RCs compared to the infectious DCs^22^. In the current study, ClpB expression was assessed by Western blot analysis using the total proteins recovered from the *E*. *chaffeensis* organisms fractionated as the top and bottom layers on renografin gradient centrifugation. ClpB expression was significantly higher in the bottom layer compared to that found in the top layer (Fig. 2A). The protein expression data for ClpB suggest that the replicating form of *E*. *chaffeensis* is fractionated at the interface of the 25–35% renografin. To further confirm the presence of DCs and RCs in top and bottom layers, respectively, cell-free organisms recovered from each of the two fractions were used to re-infect naïve Vero cells (Fig. 2B). *E*. *chaffeensis* infection in Vero cells was primarily detected when fractionated organisms from the top layer were used, but not from the bottom layer. Infectivity of fractionated *Ehrlichia* organisms from the top layer was also confirmed by judging their ability to infect HL60 cells where we estimated the percent of infected host cells (Fig. 2C). Contrary to our assumption, the presence of RC fraction in the bottom layer and DC in the top layer was puzzling, as DCs are considered more dense organisms. Therefore to further confirm this observation, we performed transmission electron microscopy to detect the presence of DC and RC forms of *E*. *chaffeensis* in top and bottom layers, respectively (Fig. 3). Indeed, the pleomorphic and larger form of *E*. *chaffeensis* is evident primarily in the bottom layer, while the condensed form of the organisms was primarily observed in the top layer. Together, the experiments confirmed that the *E*. *chaffeensis* DC form is present primarily in the renografin fractionated top layer, whereas the RC form is present in the bottom layer.

### Impact of pH and alternate energy sources on protein biosynthesis in the axenic media

**Figure 4:**
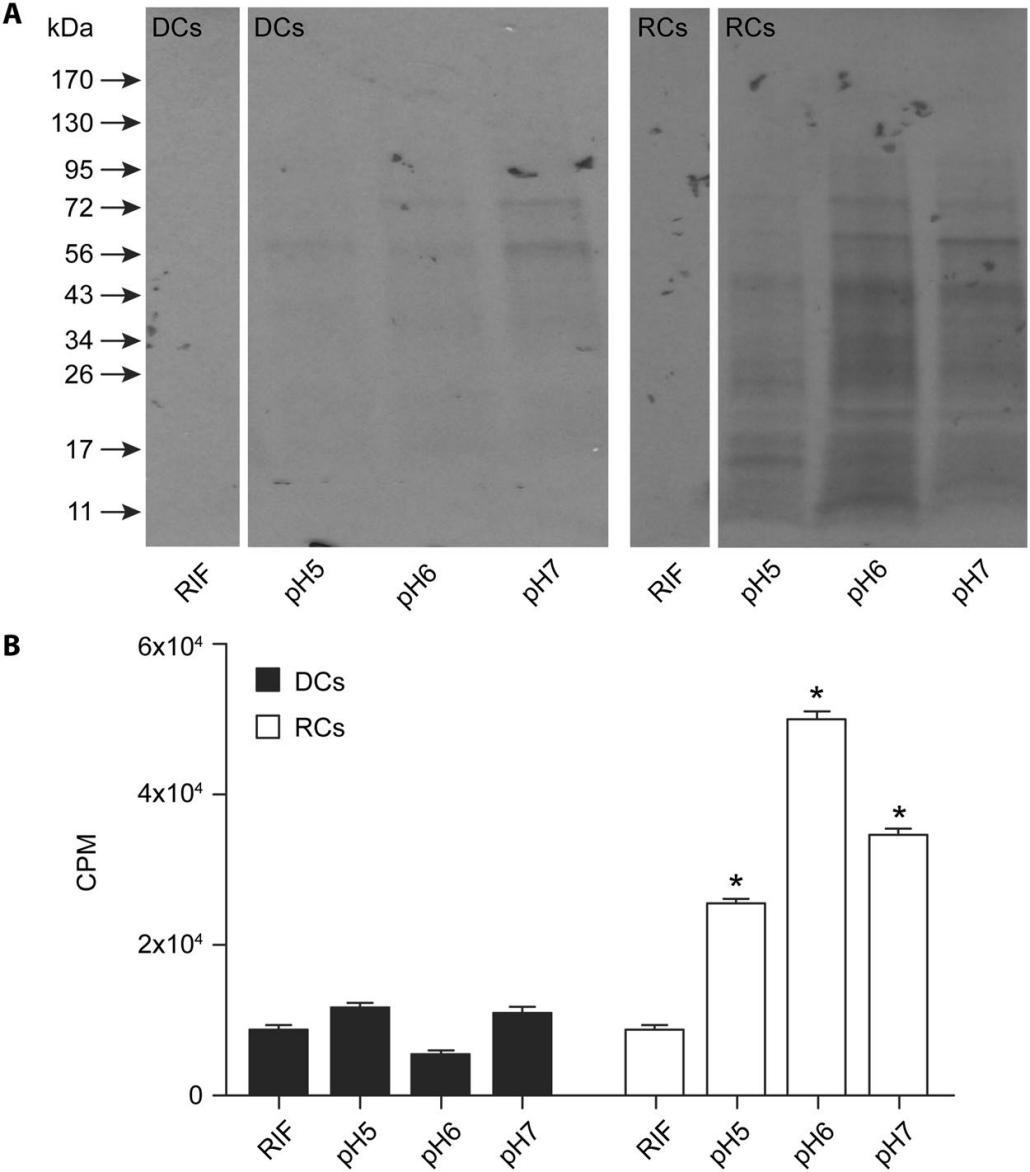
Impact of pH variations assessed on the ^35^S Cys-Met incorporation into *E*. *chaffeensis* cell-free organisms. (**A**) Autoradiography imaging was performed to assess the impact of three different pH units; 5, 6 and 7 for DCs and RCs. In the first lane, we included rifampin (RIF) containing sample with the media at pH 5.0 to serve as a negative control (**B**). As in panel **A**, except that the scintillation counting data were presented. Significant change noted relative to RIF controls are identified with a *above each bar. (Note: Original images used in preparing Figure 4A were provided as the Supplementary information file.)

**Figure 5:**
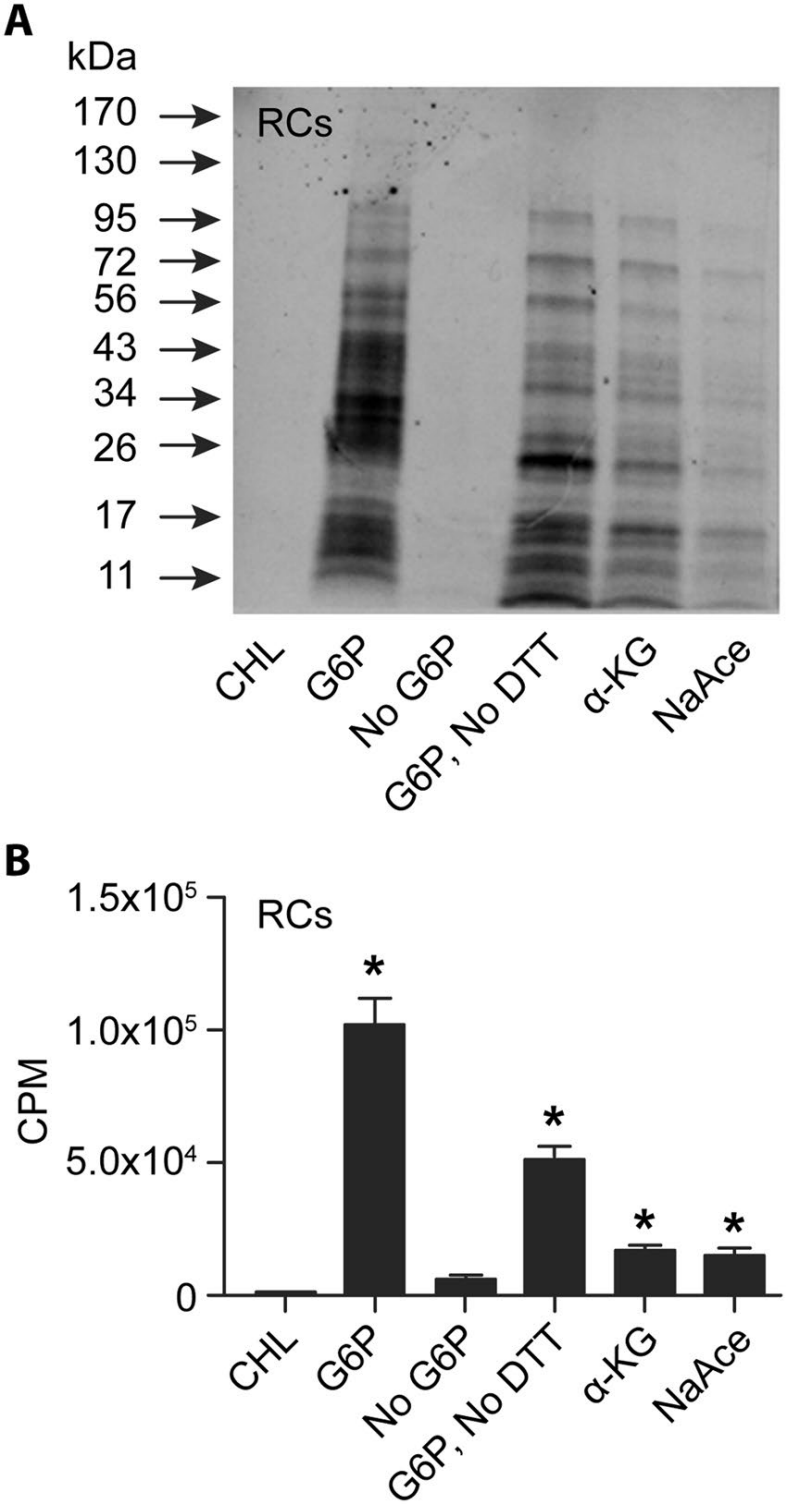
Axenic media assessment using different carbon sources for the RC fraction. (**A**) Autoradiography imaging was assessed with the axenic media containing G6P with DTT (G6P); without an energy source (no G6P); with G6P in the absence of DTT (G6P, No DTT); with α-keto glutaric acid (α-KG) or sodium acetate (NaAce) as energy sources. The first lane included axenic media with G6P and CHL to serve as the negative control. (**B**) As in panel A, but scintillation counting data were presented. Significant change noted relative to CHL control in the experimental samples are identified with a *above each bar.

We investigated if protein biosynthesis by cell-free *Ehrlichia* in the axenic media has any pH preference, and secondly, if an altered pH may promote biosynthesis by the DC form (Fig. 4). As in the previous experiment, ^35^S-Cys-Met incorporation in the RC form was significantly higher compared to the control for all three different pHs, but there was no significant difference in the levels of incorporation among the three different pHs of the medium. No significant changes in ^35^S-Cys-Met incorporation were noted for the DC form of the bacterium compared to its respective antibiotic control. We then assessed the impact of different energy sources for the cell-free protein biosynthesis. The axenic medium supplemented with G6P, α-ketoglutarate or sodium acetate supported protein biosynthesis similarly for the RC form of *E*. *chaffeensis* recovered from DH82 cultures. G6P appeared to be more favored as the carbon source, but the difference was not significant compared to other carbon sources (Fig. 5). We also tested the impact of excluding the reducing agent (DTT) in the axenic medium for protein biosynthesis. The absence of DTT had only a marginal negative effect.

### Limited protein biosynthesis confirmed by Western blot analysis

**Figure 6:**
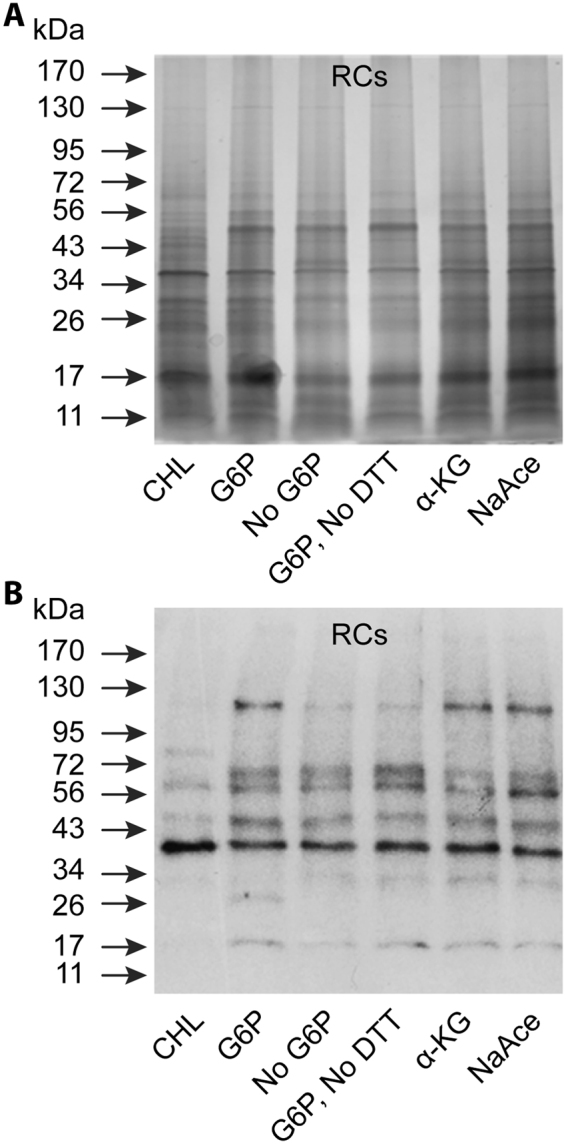
Protein biosynthesis assessed by protein fractionation and Western blot analysis. (**A**) Silver stained SDS containing polyacrylamide gel-resolved protein fractions were assessed for the protein abundance variations in RCs incubated in the axenic media with different carbon sources as in Fig. 5. (**B**) As in panel A, except that the resolved proteins were transferred to a nylon membrane and assessed by Western blot analysis using mouse polyclonal serum against *E*. *chaffeensis*.

Based on the incorporation data in the above-described experiments, we reasoned that only limited protein biosysthesis has occurred in RCs. To verify this hypothesis, we compared protein profiles of total proteins resolved on an acrylamide gel before and after assessing by Western blot analysis using antisera against *E*. *chaffeensis* (Fig. 6). Despite the presence of increased abundance of a selected sub-group of proteins when RCs were incubated in the axenic medium containing different carbon sources, increase in total proteins was only moderate compared to that observed in the axenic medium containing CHL (Fig. 6A). Consistent with these data, *Ehrlichia* immunogenic proteins, assessed using a polyclonal sera, were also moderately increased when the RCs were incubated in the axenic media (Fig. 6B).

### DNA synthesis assessed in axenic media supports the limited protein biosynthesis data

**Figure 7:**
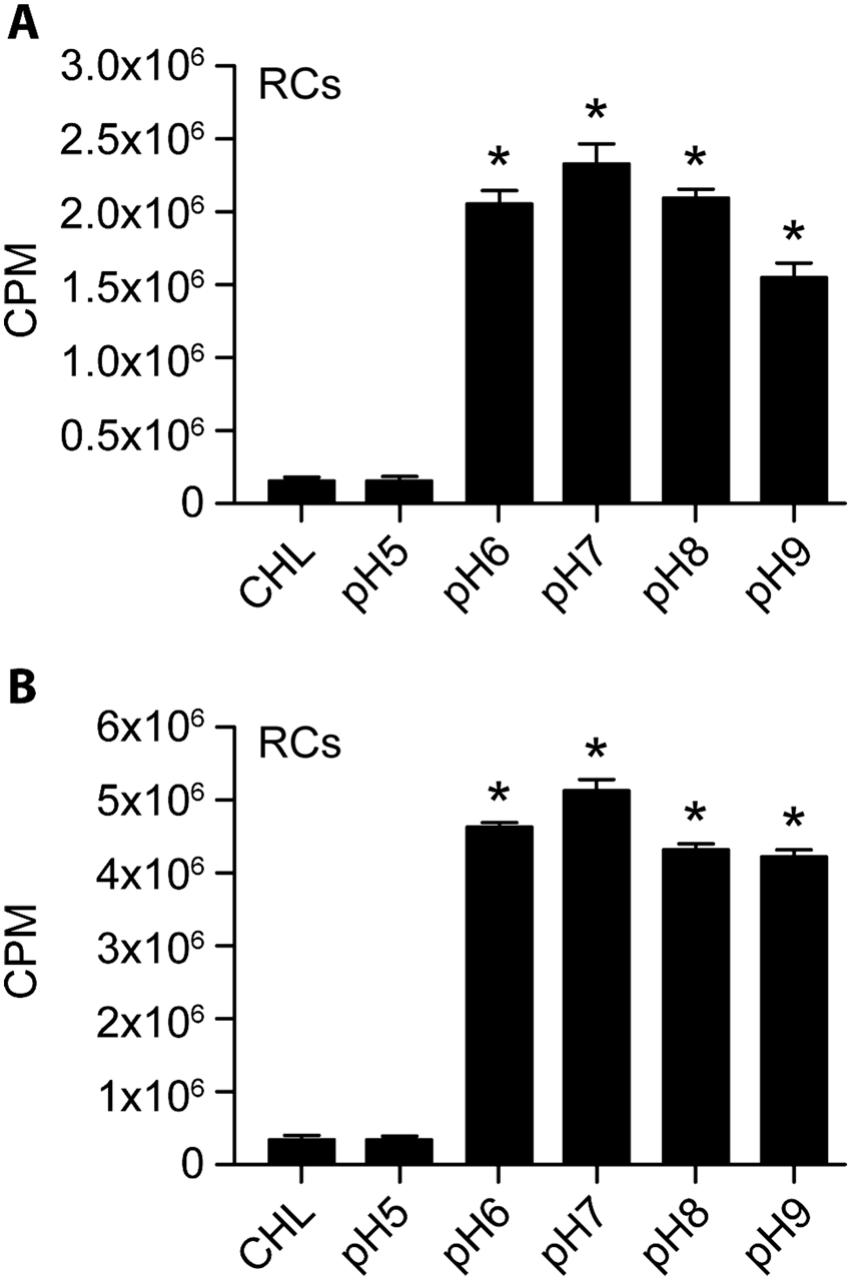
Protein biosynthesis and DNA synthesis assessed simultaneously by measuring the ^35^S Cys-Met and ^3^H thymidine incorporation, respectively in the axenic media at varying pHs of the media. (**A**) Scintillation counting data for the ^35^S Cys-Met assessed with G6P as the energy source. (**B**) As in panel A, except that the scintillation counting data for the ^3^H thymidine incorporation was assessed. Significant changes noted relative to CHL control are identified with a *above each bar.

We then tested if the axenic media also promoted DNA synthesis (Fig. 7). ^3^H thymidine incorporation was measured in the axenic media along with ^35^S Cys-Met incorporation to assess DNA and protein synthesis, respectively, for the RC form of *E*. *chaffeensis*. This assay was carried out at different pH conditions ranging from pH 5 to 9. There were no major differences in the incorporation of ^3^H thymidine or ^35^S Cys-Met when the RC form of *E*. *chaffeensis* was incubated at pH 6–9. Importantly, ^3^H thymidine incorporation in RC fraction was consistent with that of the ^35^S Cys-Met incorporation.

### RNA synthesis assessed in axenic media is consistent with DNA and protein biosynthesis

We performed a quantitative RT-PCR experiment targeting 16S rRNA using the cell-free RC fraction, as DNA and protein synthesis were observed primarily in this form of *E*. *chaffeensis*. The analysis was performed on RNA recovered from RCs incubated in the axenic media for 0 h, 2 h, 6 h, 12 h and 24 h. Triplicate RNA samples from each incubation time point were assessed by Taq-Man probe-based, quantitative real-time RT-PCR (Table 1). There was no significant change in the RNA expression level between 0 h and 2 h of incubation, while RNA copy numbers beyond 2 h of incubation resulted in a steady decline. These data suggest that the RC form of *E*. *chaffeensis* is viable only within the first two hours of incubation where protein biosynthesis and DNA synthesis occurred. The decline in RNA beyond 2 h of incubation may have resulted from continuous loss of RC viability in the axenic media. While RNA degradation is unexpected after 2 h incubation, the data are consistent with the observed moderate increase in protein and DNA biosynthesis in the RC form of *E*. *chaffeensis*.

**Table 1:** *E*. *chaffeensis* 16S rRNA assessed for the RC form following different times points of incubation in the axenic media. (* refers to significant fold change at different time points compared to 0 h value.)

Hours	Ct	SD	Fold change compared to 0 hour	*P-value
0	18.24	0.7	N/A	N/A
2	18.64	0.31	None	0.47
6	20.01	0.5	3.3	0.02^*^
12	22.87	0.15	24	0.0007^*^
24	24.64	0.22	86	0.0003^*^

## Discussion

Two major limitations of carrying out research on obligate intracellular bacterial pathogens, including the study of Rickettsiales belonging to the Anaplasmataceae family, are the lack of fully established methods for targeted mutagenesis and the inability to grow the bacteria in the absence of a host cell. Targeted mutagenesis methods aid greatly in understanding the contributions of various genes involved in pathogenesis and in defining the genes critical for the pathogens’ vector and vertebrate host cell-specific growth. However, the ability to grow the pathogens in a cell-free medium can greatly facilitate studies focused on understanding functions of various bacterial proteins without the influence of a host cell. Further, growth in a host cell-free medium will aid in rapidly recovering mutant organisms and clonally purifying mutants^9^. Indeed, recent studies on *C*. *burnetii* demonstrated that significant progress could be made with the advent of fully established methods of mutagenesis and axenic growth^14,16,23,24^. To address these two major deficiencies for the field of research on Anaplasmataceae family pathogens, we recently described methods for creating stable targeted mutations to both disrupt and also restore the function of a disrupted gene in *E*. *chaffeensis*^25^. In the present study, we focused on the second major challenge for the field: the development of an axenic culture medium to grow *E*. *chaffeensis*. We believe that the data described here are critical for moving the field forward in various fronts, while requiring making improvements to the axenic media growth method to promote the transition of the replicating form to the infectious form of *E*. *chaffeensis*. Axenic media growth conditions will be valuable for studies focused on several Anaplasmataceae pathogens; 1) to aid in identifying and characterizing effector proteins involved in influencing the host; 2) in studying the potential interactions of the bacterial phagosome with mitochondria, host cytoplasmic proteins and nucleus; and 3) in facilitating the clonal purification of mutated organisms resulting from random and targeted mutagenesis methods.

Firstly, we presented a method for purification of *E*. *chaffeensis* DCs and RCs from host cells by employing renografin density gradient centrifugation. We discovered that the DC form of the bacterium fractionated at a lower concentration of renografin compared to the RC form. The presence of DCs and RCs within the gradient fractions was confirmed by three independent methods: the ability to infect naïve host cells, morphology, and by protein expression. Our studies demonstrate that axenic media supports protein and DNA synthesis only in the RCs of *E*. *chaffeensis*. Our study also provides definitive proof that the DC form is the infectious form, while the RC form is a pleomorphic replicating form that is actively involved in protein and DNA synthesis. Axenic media-specific protein synthesis was further confirmed by inclusion of the inhibitors, chloramphenicol or rifampin, in the cell-free media.

Axenic media-specific protein synthesis in *E*. *chaffeensis* is similar to a prior study demonstrating the cell-free protein biosynthesis for *C*. *trachomatis*^20^. In the current study we presented evidence that the axenic media also supported bacterial DNA synthesis in *E*. *chaffeensis*. Despite protein and DNA synthesis shown in the absence of host cells for RCs, our data suggest that the abundance is limited. We further investigated if variations in the pH of the media and altered energy sources may improve the protein biosynthesis. G6P appeared to be the best energy source for RC fraction. We did not note any conditions in the axenic media with the capacity to promote protein synthesis in DC fraction of *E*. *chaffeensis*.

**Figure 8:**
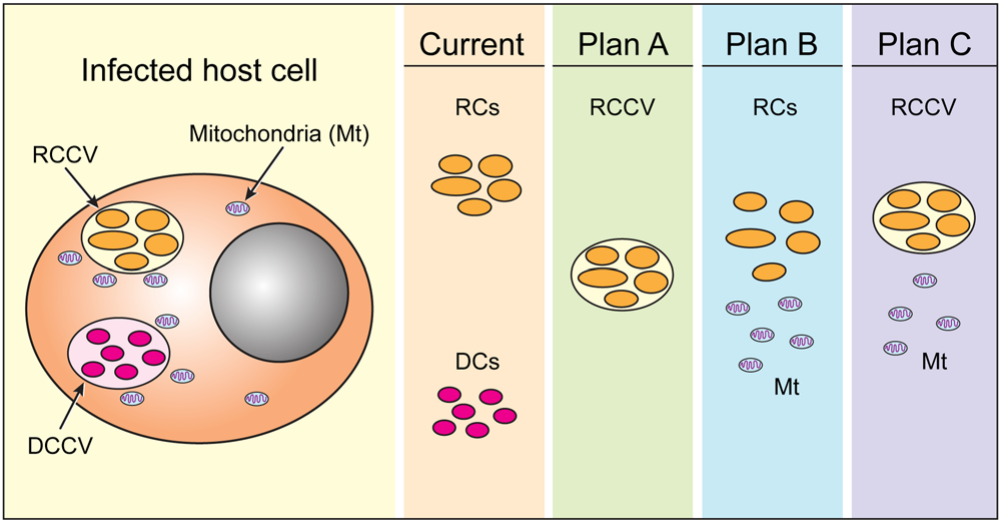
Proposed model to make improvements to the axenic media to promote *E*. *chaffeensis* replication *in vitro* in the absence of a host cell. RCCV and DCCV refer to RC containing and DC containing phagosome vacuoles, respectively.

Two important goals to improve the axenic media for *E*. *chaffeensis* are to modify the media conditions, such as adding thymidine in the media cocktail to 1) promote increased DNA and protein synthesis, resulting in the continued replication of the RC form; and 2) to transform the RCs to DCs under axenic media conditions. These improvements may be possible if the axenic media growth is assessed with purified, host cell-derived *E*. *chaffeensis* RC-containing phagosomes in place of purified RCs (Fig. 8). We reasoned that the phagosomal microenvironment might mimic *in vivo* conditions, although it may limit the number of bacterial replications. Axenic growth may also be further improved with the addition of purified mitochondria to the media containing the host cell-free RC fraction or RC-containing phagosomes (Fig. 8). Prior studies by us and other investigators focused on transmission electron microscopy^11–13^ demonstrated that mitochondria are closely associated with *E*. *chaffeensis-*containing phagosome vacuoles of infected host cells. *E*. *chaffeensis* and other Anaplasmataceae pathogens may benefit from mitochondria in multiple ways, including obtaining energy and metabolites.

In summary, the data presented here represent a significant step in advancing the goal of developing axenic media growth of *E*. *chaffeensis*. While the method needs improvement, we believe the strategy used in the current study can be adapted to other important pathogens belonging to Anaplasmataceae pathogens belonging to the genera *Ehrlichia*, *Anaplasma* and *Neorickettsia*.

## Materials and Methods

### Cultivation of *E. chaffeensis*

*E*. *chaffeensis* was cultivated in canine macrophage cell line, DH82 as described previously^26^. Similarly, *E*. *chaffeensis* in Vero cells (ATCC, Manassas, VA) was cultured in the complete MEM medium (Thermo Fisher Scientific, Waltham, MA) supplemented with 7% fetal bovine serum (Thermo Fisher Scientific, Waltham, MA) and 2 mM L-glutamine (Mediatech, Manassas, VA). Cultivation of *E*. *chaffeensis* in HL60 cells (ATCC, Manassas, VA) was in complete RPMI 1640 medium (Thermo Fisher Scientific, Waltham, MA) supplemented with 10% fetal bovine serum (Thermo Fisher Scientific, Waltham, MA) and 2 mM L-glutamine (Mediatech, Manassas, VA) by following the protocols described elsewhere for *Anaplasma phagocytophilum*^27^. To prepare cell-free *Ehrlichia* inocula, about 80–100% *E*. *chaffeensis*-infected DH82 cells in a T25 flask were harvested by centrifugation at 400 × g for 10 min at 4 °C. The pellets were resuspended in 5 ml of serum-free medium, and the cells were disrupted with glass beads by vortexing twice for 30 sec. The cell debris and unbroken cells were removed by centrifugation at 200 × g for 10 min at 4 °C. The supernatant was passed through a 2.7 µm pore-size syringe filter (Whatman, Pittsburgh, PA). HL60 cells were incubated with host cell-free *E*. *chaffeensis* (about 100 bacteria per host cell) for 120 min to allow for internalization. Non-ingested *E*. *chaffeensis* were removed by washing with PBS, and the cells were incubated for an additional 3 days in T150 flasks. Similar infection protocol is followed when infecting Vero cells or DH82 cells. When the infectivity reached to 80–90%, the infected host cell cultures were harvested by centrifugation at 15,000 × g for 5 min at 4 °C and used for purifying the host cell-free bacteria, as outlined below.

### Purification of *E. chaffeensis*

*E*. *chaffeensis* organisms in the forms of dense-core cells (DCs) and reticulate cells (RCs) were purified by renografin density gradient centrifugation as described previously^28,29^ with some minor modifications. In brief, pellets of infected host cells were suspended in sterile PBS. The cells were then homogenized at 4 °C using a 10 ml syringe with a 23^1/2^-G needle; typically 10–15 strokes were used to disrupt the cells. Homogenization was carried out until approximately 90% of cells were disrupted without major breakage of nuclei, as monitored by light microscopy. The disrupted cell suspension was centrifuged at 500 × g for 5 min at 4 °C. The supernatant was collected and filtered through 2.7 µm sterile syringe filter. The filtered supernatant was then centrifuged at 15,000 × g for 15 min at 4 °C. The pellet was resuspended into sterile PBS and 2 mL of the suspension was layered over discontinuous renografin gradients (3 mL 25%, 4 mL of 35% renografin in PBS, vol/vol). These gradients were centrifuged at 100,000 × g for 1 h at 4 °C using a Swinging Bucket rotor (S50-ST) in a Sorvall MTX150 ultracentrifuge (Waltham, MA). Fractions at the interfaces of PBS-25% and 25–35% renografin were collected using a sterile syringe, diluted with three volumes of PBS, and then centrifuged at 15,000 × g for 15 min at 4 °C. The pellets were washed with PBS to remove residual renografin by repeating the centrifugation step 15,000 × g for 15 min at 4 °C, and then the final purified pellets were resuspended in PBS for use in the cell free activity experiments.

### Preparation of axenic medium

The axenic medium was prepared according to the previous study on *C*. *trachomatis* cultured in axenic medium as per the compositions and concentrations of each component^20^. Depending on the experiment carried out, the medium contained or excluded glucose 6-phoshate (G6P) or adenosine triphosphate (ATP) or alpha ketoglutarate or sodium acetate to serve as carbon sources. Similarly, pH of the media is modified as per the experimental need.

### Protein synthesis by ^35^S-cysteine–methionine incorporation

Protein synthesis in cell-free purified fractions of *E*. *chaffeensis* was measured by incorporation of ^35^S-Cys-Met (Perkin Elmer, Waltham, MA) as described previously^20^. For normalization of bacterial total protein content, the suspensions of *E*. *chaffeensis* cell-free fractions were lysed in 1% SDS solution for 5 min at 100 °C and the total protein concentration was determined using Protein Assay kit (Bio-Rad, Hercules, CA). Subsequently, the suspensions of *E*. *chaffeensis* cell-free fractions were equally split into micro-centrifuge tubes at the amount of 30 µg total proteins. Partially opened micro-centrifuge tubes containing 500 μL of media supplemented with 70 µCi of ^35^S-Cys-Met were incubated at 37 °C for 24 h in a tri-gas humidified incubator set to maintain 2.5% O_2_. *E*. *chaffeensis* cell-free organisms were pelleted at the end of incubation by centrifugation at 15,000 × g for 15 min at 4 °C, washed with K-36 buffer (0.05 M K_2_HPO_4_, 0.05 M KH_2_PO_4_, 0.1 M KCl, 0.15 M NaCl, pH7.0) twice, and disrupted by adding 30 µL of 2 × SDS-PAGE sample buffer and by boiling for 5 min^20^. Ten µL of lysate each was then transferred to a tube containing 5 mL of biosafe liquid II and used for quantification of ^35^S-Cys-Met incorporation using the protocol 4 (^35^S) in a liquid scintillation counter (TRI-CARB 2100TR, PerkinElmer, Waltham, MA). For visualizing the radiolabel incorporation into bacterial proteins, equal volumes of sample lysates were also separated in an SDS/PAGE and the gel was dried and exposed to an X-ray film. Similarly, cell-free growth experiments were carried out in the absence of ^35^S-Cys-Met, resolved on an SDS-PAGE gel, and stained using silver nitrate staining kit (Thermo Fisher Scientific, Waltham, MA) as per the manufacturer’s recommendations.

### DNA synthesis by ^3^H-thymidine incorporation

Purified *E*. *chaffeensis* cell-free fractions were also assessed for incorporation of ^3^H-thymidine (Perkin Elmer, Waltham, MA) into the bacterial DNA simultaneously with the incorporation ^35^S-Cys-Met into proteins^30^. Briefly, *E*. *chaffeensis* cell-free organisms were incubated for 48 h at 37 °C with 2.5% O_2_ in micro-centrifuge tubes containing 500 µL of medium supplemented with 20 μCi of ^3^H-thymidine and 70 μCi of ^35^S-Cys-Met. *E*. *chaffeensis* were pelleted at 15,000 × g for 15 min at 4 °C, washed with K-36 twice, lysed in 30 µL of 2 × SDS-PAGE sample buffer and then boiled for 5 min. 10 µL of lysate each was added into 5 mL of Biosafe liquid II (Grainger, Hartford, CT) and used for quantification of ^3^H-thymidine incorporation (the protocol 10, ^3^H) and ^35^S-Cys-Met incorporation (the protocol 4, ^35^S) using a liquid scintillation counting machine (TRI-CARB 2100TR, PerkinElmer, Waltham, MA), respectively.

### Sodium dodecyl sulfate-polyacrylamide gel electrophoresis

Five μL of NuPAGE SDS sample buffer and 2 μL of NuPAGE reducing agent (Invitrogen/Thermo Fisher Scientific, Waltham, MA) were added to each of 10 μL of sample solution following cell-free incubation experiments in the axenic medium, boiled for 5 min, and then loaded onto a Mini-PROTEAN Precast Bis-Tris 4% to 14% polyacrylamide gels (Bio-Rad, Hercules, CA) and subjected to electrophoresis (100 mA/gel for 60 minutes). The gels were then recovered from the gel assembly and stained using a Silver staining kit (Thermo Fisher Scientific, Waltham, MA) according to the manufacturer’s recommendations.

### Western blot analysis to assess protein synthesis

For the detection of ClpB protein of *E*. *chaffeensis*, the above-described electrophoresed proteins were transferred onto a nitrocellulose membrane (Thermo Fisher Scientific, Waltham, MA) by subjecting to electro-blotting using an electrophoretic transfer unit (Bio-Rad, Hercules, CA). Protein transfer buffer was prepared as per the manufacturer’s instructions and used in the protein transfer protocols. Subsequently, *E*. *chaffeensis* ClpB expression was assessed using polyclonal rabbit antisera raised against recombinant *E*. *chaffeensis* proteins for ClpB, respectively^22^. Secondary anti-rabbit antibody conjugated with horseradish peroxidase (Sigma-Aldrich, St. Louis, MO, USA) and Super Signal West Pico Chemiluminescent Substrate (Thermo Fisher Scientific, Waltham, MA) were used for the signal detection, respectively. We also performed similar blot analysis using polyclonal sera generated in the murine host in response to *E*. *chaffeensis* infection^31^.

### Quantitative real-time RT-PCR to measure *E*. *chaffeensis* 16S rRNA expression

Cultures of *E*. *chaffeensis* grown in several T150 flasks were used in recovering cell-free RC form of the organisms fractionated on a renografin density gradient centrifugation as described above. *E*. *chaffeensis* RC organisms in triplicate microcentrifuge tubes were incubated for 0 h, 2 h, 6 h, 12 h and 24 h with 500 µL of axenic medium containing G6P and ATP at 37 °C with 2.5% O_2_. At the end of the specified incubation times, cells were recovered by centrifugation at 15,000 × g for 10 min at 4 °C. The bacterial pellets were then inactivated immediately in the TRI reagent solution, and then used to isolate total RNA by TRI reagent protocol (Sigma-Aldrich, St. Louis, MO). Final recovered RNA from each tube was resuspended in 25 μl of nuclease-free water, then DNase treated to remove residual genomic DNAs using RQ1 DNase (Thermo Fisher Scientific, Waltham, MA). RNA from each tube was diluted 1:1000 in nuclease-free water and 2 μl each was used in 25 μl reaction in performing Taq-Man probe-based real-time RT-PCR targeted to the *E*. *chaffeensis* 16S RNA as previously described^32^. The RNA levels in each sample were expressed by Ct values. Variation among triplicates for each time point was calculated and presented with the respective standard deviations observed. Fold changes were calculated relative to the Ct values observed for the RNA recovered before incubation (0 h) compared to different incubation times. The data were then assessed for statistical significance.

### Preparation of *E*. *chaffeensis* cultures for use in transmission electron microscopy

Purified *E*. *chaffeensis* DCs and RCs were resuspended in PBS and used in transmission electron microscopy analysis by following the methods described previously^12^. Briefly, all TEM preparation steps were followed by repelleting samples by centrifugation at 4 °C for 5 min at 200 × g, unless otherwise specified. Pelleted DCs and RCs (in 10 μl volume) were fixed with 0.5 ml of Karnovsky’s fixative containing 2% paraformaldehyde, 2.5% gluteraldehyde in 0.1 M cacodylate buffer (pH7.4) overnight at 4 °C. The cell-free *E*. *chaffeensis* organisms were then washed three times with 1 ml of 0.1 M cacodylate buffer, post-fixed in 2% osmium tetroxide in the same cacodylate buffer, washed, enblock stained in 1% aqueous uranyl acetate for 1 h, washed, dehydrated in a 50–100% ascending series of acetone, infiltrated and embedded in Spurr’s resin. Ultrathin sections (<95 nm thick silver to gold) were cut using ultramicrotome, examined with a CM 100 TEM (FEI Company, Hillsboro, OR, USA now Thermo Fisher Scientific), and images captured with a Hamamatsu C8484 digital camera using a AMT digital image capture system (Chazy, NY).

### Statistics analysis

Differences in quantitative protein, DNA synthesis and RNA expression between groups were assessed using Student’s t-test using the online software (http://www.socscistatistics.com/tests/studentttest/Default.aspx), with significance considered for p < 0.05.

## Electronic supplementary material

Supplementary Information
