# Supplementary material for: Protein and DNA synthesis demonstrated in cell-free Ehrlichia chaffeensis organisms in axenic medium
Source: Sci Rep. 2018 Jun 18;8:9293. doi: 10.1038/s41598-018-27574-z (PMC6006305; doi:10.1038/s41598-018-27574-z)
Supplement: Supplementary file 1 — Supplementary Information [file 41598_2018_27574_MOESM1_ESM.pdf]

Protein and DNA synthesis demonstrated in cell-free *Ehrlichia chaffeensis* organisms in axenic medium

Vijay K. Eedunuri[1#§], Yuntao Zhang[1#], Chuanmin Cheng[1§§], Li Chen[1], Huitao Liu[1], Anders Omsland[2], Dan Boyle[3], and Roman R. Ganta[1]*

[1]Center of Excellence for Vector-Borne Diseases, Department of Diagnostic Medicine/Pathobiology, College of Veterinary Medicine, Kansas State University, Manhattan, KS 66506; [2]Paul G. Allen School for Global Animal Health, PO Box 647090, Washington State University, Pullman WA 99164; [3]Division of Biology, Kansas State University, Manhattan, KS 66506

[#]These two authors contributed equally to the manuscript

[§]Current address; UT Health Science Center at San Antonio, San Antonio, TX; [§§] Vanderbilt University Medical Center, Nashville, TN

*Corresponding author

Original images used in making Figure 4 (top panel):

Figure 4; A, DCs (top left section)

DCs

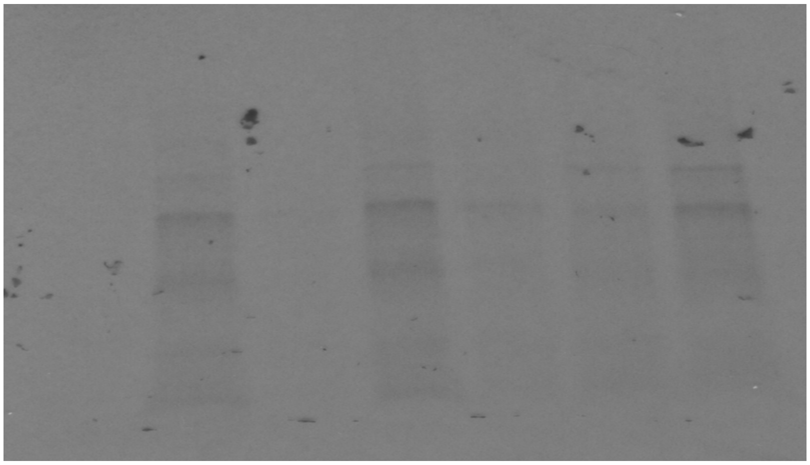

RIF pH5 pH6 pH7

Figure 4; A, RCs (top right section)

RCs

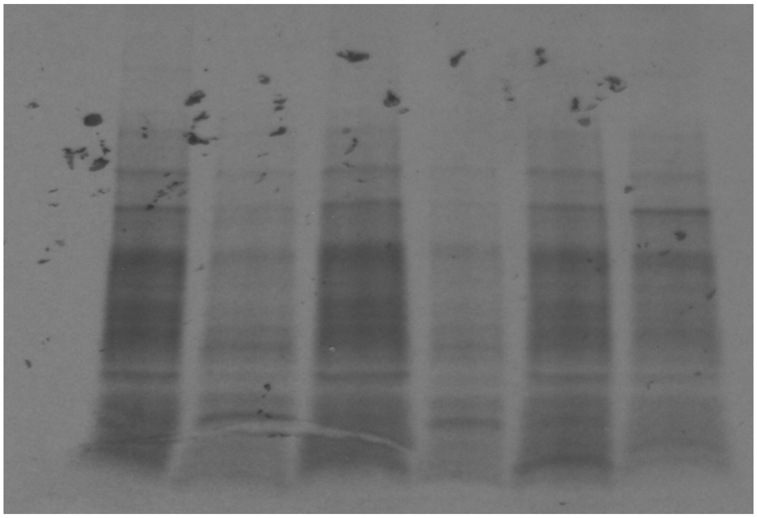

RIF pH5 pH6 pH7
